# Whole-Genome Sequencing of Alcaligenes faecalis HZ01, with Potential to Inhibit Nontuberculous Mycobacterial Growth

**DOI:** 10.1128/MRA.00521-21

**Published:** 2021-09-30

**Authors:** Henry Marcel Zalona Fernandes, Emilyn Costa Conceição, Sandro Patroca da Silva, Edson Machado, Maria Carolina Sisco, Abhinav Sharma, Karla Valéria Batista Lima, Marília Lima da Conceição, Ana Carolina da Silva Carvalho, Karla Rodrigues Miranda, Rafael Silva Duarte, Daniela Sales Alviano, Rubens Clayton da Silva Dias

**Affiliations:** a Instituto de Microbiologia Paulo de Góes, Universidade Federal do Rio de Janeiro, Rio de Janeiro, Rio de Janeiro, Brazil; b Department of Science and Innovation, National Research Foundation Centre of Excellence for Biomedical Tuberculosis Research, South African Medical Research Council Centre for Tuberculosis Research, Division of Molecular Biology and Human Genetics, Faculty of Medicine and Health Sciences, Stellenbosch University, Cape Town, South Africa; c Programa de Pós-graduação em Pesquisa Clínica e Doenças Infecciosas, Instituto Nacional de Infectologia Evandro Chagas, Fundação Oswaldo Cruz, Rio de Janeiro, Rio de Janeiro, Brazil; d Instituto Evandro Chagas, Ananindeua, Pará, Brazil; e Laboratório de Genética Molecular de Microrganismos, Instituto Oswaldo Cruz, Fundação Oswaldo Cruz, Rio de Janeiro, Rio de Janeiro, Brazil; f Faculty of Engineering and Technology, Liverpool John Moores University, Liverpool, United Kingdom; g Pós-Graduação Biologia Parasitária na Amazônia, Instituto de Ciências Biológicas e da Saúde, Universidade do Estado do Pará, Belém, Pará, Brazil; h Instituto de Química, Universidade Federal do Rio de Janeiro, Campus Macaé, Macaé, Rio de Janeiro, Brazil; i Instituto Biomédico, Universidade Federal do Estado do Rio de Janeiro, Rio de Janeiro, Rio de Janeiro, Brazil; Loyola University Chicago

## Abstract

Alcaligenes faecalis is a Gram-negative rod that is ubiquitous in the environment and is an opportunistic human pathogen. Here, we report the whole-genome sequencing analysis of A. faecalis HZ01, which presents mycobacterial growth inhibitory activity and was isolated from a contaminated culture of Mycobacterium chubuense ATCC 27278.

## ANNOUNCEMENT

Alcaligenes faecalis is a Gram-negative rod, nonfermenting, aerobic, mobile, and peritrichous bacterium (1). This opportunistic pathogen is widely distributed in the environment and is related to nosocomial diseases (2, 3), with biotechnological potential in the pharmaceutical industry and in bioremediation of contaminated environments (4), such as the production of antibacterial substances (5–7). Although A. faecalis represents a promising source for new bioactive substances, there is limited literature on genomic approaches (8).

**FIG 1:**
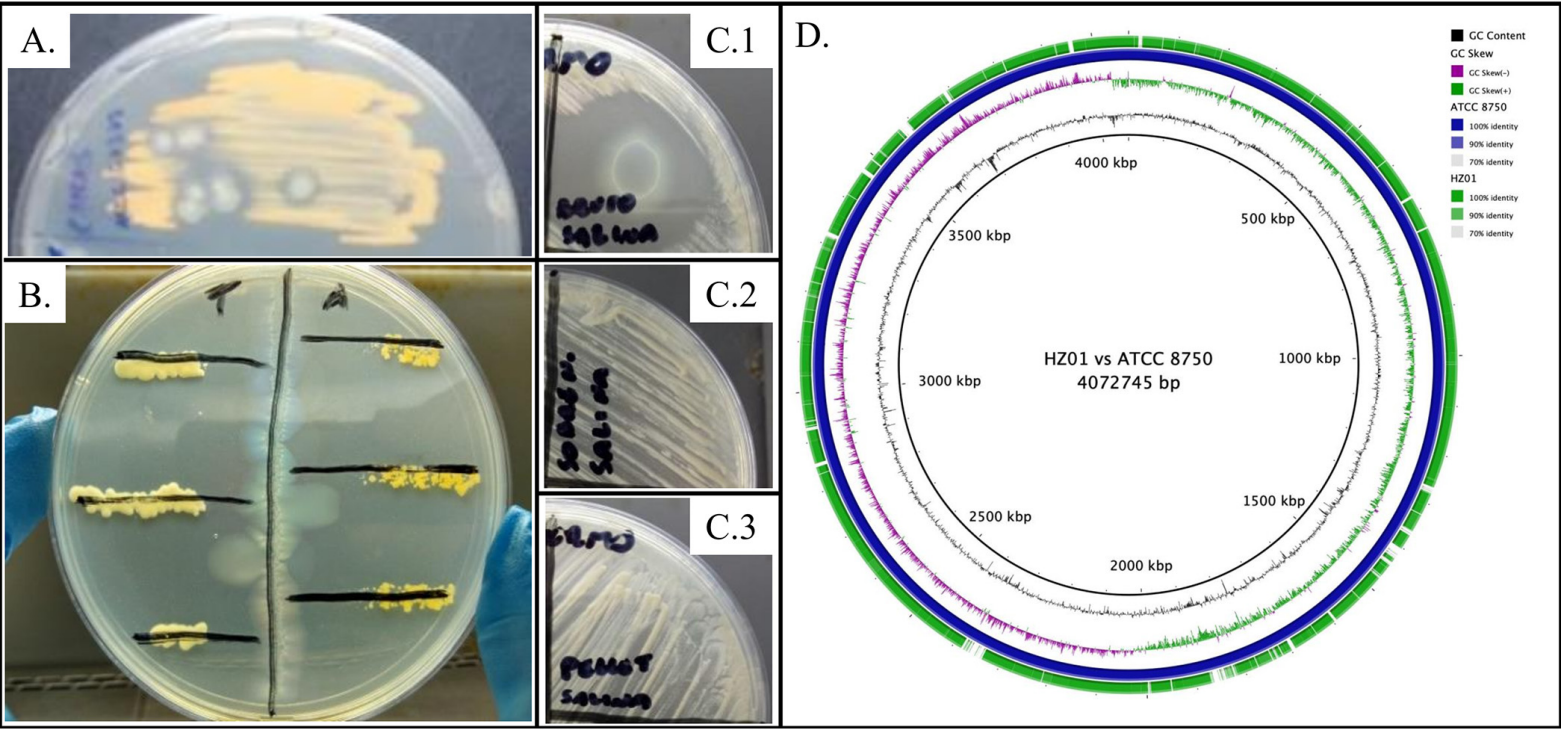
Antimycobacterial activity of Alcaligenes faecalis HZ01. (A) Mycobacterium chubuense ATCC 27278 culture from which A. faecalis HZ01 was originally isolated. (B) Antibiosis test exhibiting A. faecalis HZ01 (central line) antimycobacterial activity on Mycobacterium thermoresistibile ATCC 19527 (lines on the left) and Mycobacterium aichiense ATCC 27280 (lines on the right), using the cross-streak method. (C) Antimycobacterial activity analysis of A. faecalis HZ01 cell suspension (C.1), cell-free supernatant (C.2), and lysed pellet (C.3) on a M. thermoresistibile ATCC 19527 culture. (D) Genomic comparison of the A. faecalis HZ01 isolate against the reference genome of A. faecalis ATCC 8750.

During the development of previous studies, we observed a contaminant microorganism that had grown on a Mycobacterium chubuense ATCC 27278 culture at 37°C on Middlebrook 7H10 medium and exhibited mycobacterial growth inhibitory activity (Fig. 1A to C). In a similar study, it was verified that the antibacterial activity of A. faecalis is via a live-cell and contact-dependent mechanism (9). The ATCC strain was obtained from our mycobacterial collection. To isolate the contaminant microorganism, we selected three colonies showing a halo of mycobacterial growth inhibition, and then they were individually streaked on another Middlebrook 7H10 medium plate and incubated at 37°C for 48 h. The contaminant microorganism was identified as A. faecalis by matrix-assisted laser desorption ionization–time of flight mass spectrometry (MALDI-TOF MS) of pure cultures of the three isolates obtained originally and was stored at −80°C in nutrient broth supplemented with glycerol (final concentration of 15% [vol/vol]) (10).

Following the bacterial culture in MacConkey agar in a 37°C incubator for 48 h, we performed genomic DNA extraction using the QIAamp DNA minikit (Qiagen, Hilden, Germany) and library preparation using the Nextera XT DNA library preparation kit (Illumina, San Diego, CA, USA). Whole-genome sequencing (WGS) was conducted on the Illumina NextSeq 500 platform with 2 × 150-bp paired-end reads.

The sequencing quality was evaluated using FastQC v0.11.9 (11), before and after the reads were trimmed with Trimmomatic v0.39 (12). *De novo* assembly was performed with SPAdes v3.14.0 (13), assembly quality was evaluated with QUAST v5.0.2 (14), and annotation was performed with the NCBI Prokaryotic Genome Annotation Pipeline (PGAP) v5.2 (15). For genome comparison, the Artemis Comparison Tool and BLAST Ring Image Generator (BRIG) v3.0 were used (16, 17). For variant calling, we used Snippy v4.6.0 (https://github.com/tseemann/snippy). We used PlasmidSeeker v1.3 and PlasmidFinder v2.1 to investigate the presence of plasmids (18, 19). We used default parameters for all software.

A total of 8,369,218 reads were obtained, and the genome coverage was 606×. We obtained a total of 7,854,398 reads after quality trimming. By mapping the reads obtained against A. faecalis subsp. *faecalis* (ATCC 8750) (https://genomes.atcc.org/genomes/a6829cff570e4f50) using the Burrows-Wheeler aligner (20), we observed that 86.68% of the reads were properly paired against the reference genome. After *de novo* assembly, we obtained 57 contigs; the largest contig had 848,880 bp. The draft genome obtained had a total length of 4,141,412 bp, with a GC content of 56.79% (Fig. 1D). The *N*_50_ and *N*_75_ values were 669,949 bp and 410,060 bp, respectively. There was no presence of plasmids. We found 7,873 complex variants, 191 deletions, 188 insertions, 993 multiple-nucleotide polymorphisms (MNPs), 4,7401 single-nucleotide polymorphisms (SNPs), and a total of 5,6647 variants.

Due to increasing challenges in treating multidrug-resistant infections, such as mycobacterial diseases, and the global shortage of successful drug therapy options, the discovery of new antimicrobial agents is necessary to improve patient outcomes.

### Data availability.

The A. faecalis HZ01 WGS data were deposited in DDBJ/ENA/GenBank under accession number JAFMOE000000000 (the version described in this paper is JAFMOE010000000), BioSample accession number SAMN17762316, BioProject accession number PRJNA698913, and SRA accession number SRR13612681.
